# Adaptation of *Betula pendula* Roth., *Pinus sylvestris* L., and *Larix decidua* Mill. to environmental stress caused by tailings waste highly contaminated by trace elements

**DOI:** 10.1007/s10661-023-12134-4

**Published:** 2023-12-18

**Authors:** Bartłomiej Świątek, Wojciech Kraj, Marcin Pietrzykowski

**Affiliations:** 1 Department of Ecological Engineering and Forest Hydrology, Faculty of Forestry, University of Agriculture in Kraków, Al. 29 Listopada 46, 31–425, Krakow, Poland; 2 Department of Forest Ecosystem Protection, Faculty of Forestry, University of Agriculture in Kraków, Al. 29 Listopada 46, 31–425, Krakow, Poland

**Keywords:** Heavy metals, Cadmium, Pine, Larch, Birch

## Abstract

The seedlings of some tree species can successfully develop in areas polluted by heavy metals. Research on such species is important in order to explore the possibility of introducing tree species for the permanent biological stabilization and reclamation of post-flotation tailings, especially after the final recycling of trace metals, but where concentrations remain much higher than in natural soils. To better understand the adaptation and reaction of *Betula pendula* Roth., *Pinus sylvestris* L., and *Larix decidua* Mill. seedlings to heavy metals pollution caused by tailings waste highly contaminated by trace elements: 1) the relationships between the concentration of heavy metals in the soil substrate, the efficiency of heavy metal ions accumulation in plant organs, and the biometric parameters of the seedlings; and 2) the threshold content of heavy metals in the roots above which the plant physiological response is triggered was determined. We assume that there are certain limit concentrations of heavy metals in the soil and fine roots, which depend on the tree species and beyond which the plant responds strongly to stressThe obtained results showed that *Betula* is a suitable species for the phytostabilization of post-flotation tailings due to its rapid growth rate and production of root biomass. The accumulation of metals in *Betula* roots was found to be much greater than in Pinus and *Larix*. Despite the high concentrations of heavy metals in the prepared substrates, there was only a slight transfer of these elements to the aboveground parts of the plant. At high soil concentrations, the heavy metals adversely affected the cellular and physiological processes of plants. In plants growing in such conditions, the activity of the antioxidant system depended both on the species and organ of the plant, as well as on the type and metal concentration.

## Introduction

Mining activity produces significant amounts of waste materials, causing a disruptive and negative impact on the environment (Hoskin, 2000). Mining and flotation tailings related to the exploitation of non-ferrous metal ores are a source of heavy metal pollution, especially due to the transport of post-flotation tailings and the erosion of landfill (Conesa et al., 2007; Li, 2006; Wang et al., 2009). Aeolian erosion, which intensifies in periods of low precipitation, is an unfavorable factor that poses a significant threat to the environment. Forests in the vicinity of tailings ponds show signs of pollution and their growth is clearly weakened. The reclamation of post-flotation tailings ponds is extremely difficult due to the high concentration of metals contained in them, alongside their low nutrient levels, high pH values, and the low water retention capacity of the soil (Cano-Reséndiz et al., 2011; Conesa et al., 2007; Krzaklewski & Pietrzykowski, 2002; Ye et al., 2002). The lack of nutrients, or their low availability, is an important selection factor that limits the development of vegetation (Kazakou et al., 2008; Turnau et al., 2010). Most post-flotation tailings pond landfills are devoid of vegetation cover, and the natural emergence of pioneer plants is quite exceptional (Monterroso et al., 2014). The problems of nutrient deficiency and lack of vegetation can be solved by covering the post-flotation tailings with a top layer of fertile soil, which must often be transported over long distances, significantly increasing costs, especially in the case of large tailings ponds (Kasowska et al., 2018). Because post-flotation waste is a potential source for the recycling and recovery of non-ferrous metals, tailing ponds are not subject to full reclamation, rather only temporary revegetation and technical and biological stabilization (Cheng et al., 2015; Krzaklewski & Pietrzykowski, 2002). An environmentally friendly and inexpensive technology is phytoremediation, which uses plants to neutralize pollutants without extracting them (Novo et al., 2013). The most important factor in phytoremediation technology is the appropriate selection of plants characterized by rapid growth, high biomass gain, high tolerance to excess metals, and intensive growth in conditions of nutrient deficiency (Deng et al., 2007; Pottier et al., 2015). Within the pool of species that can be used in phytoremediation, trees are highly effective in stabilizing heavy metals (Mendez & Maier, 2008).

Due to the participation of many heavy metals in redox reactions, and their function as cofactors or enzyme activators, the tolerance range of plants toward metals varies depending on the tree species, type, concentration, and chemical form of the metal, as well as the composition and pH of the soil. Exceeding the limit values of their concentrations negatively affects the growth of trees and is toxic to cells, tissues, and entire plants (Asgari Lajayer et al., 2017, 2019; Chandel et al., 2023; Dimkpa et al., 2009; Kraj & Zarek, 2021). However, there is a group of metal ions such as cadmium (Cd), mercury (Hg), lead (Pb), and arsenic (As) that are biologically irrelevant and highly toxic even at low concentrations in various biochemical and physiological processes (Asgari Lajayer et al., 2017, 2019; Hristozkova et al., 2016). Heavy metals are therefore an important stress factor (Fryzova et al., 2018). Elements such as Cd and Pb are trace elements and have no nutritional or physiological function in plants (Schützendübel & Polle, 2001). Due to its high mobility in the soil–plant system, Cd has been classified as the fourth most toxic element to higher plants (Qayyum et al., 2017). Heavy metals and salinity are examples of abiotic stress factors, a common effect of which (in addition to limiting shoot and root growth) is an imbalance between the generation and capture of potentially cell-damaging reactive oxygen species (ROSs). The accumulation of ROSs leads to oxidative stress (Sidhu et al., 2017). The emerging imbalance of the redox state in cells is caused, on one hand, by an increase in the content of superoxide ions (O_2_•–), hydrogen peroxide (H_2_O_2_), and thiobarbituric-acid-reactive substances (TBARSs)––an indicator of membrane lipid peroxidation. This results from the activation of oxidases located in the membranes and the interruption of the electron transport chain in the photosynthetic system in chloroplasts and the respiratory chain in the mitochondria, causing oxidation and inhibition of the activity of cellular proteins. On the other hand, based on the lower ability to synthesize small molecule antioxidants (primarily ascorbic acid and glutathione), the decreasing activity of antioxidant enzymes such as superoxide dismutase, catalase, and enzymes of the Foyer-Halliwell-Asada cycle, plants show lower activity of the cellular antioxidant system (Abbas et al., 2017; Yongqi et al., 2016). Plants protect themselves against the adverse effects of ROSs by increasing the synthesis and activity of small-molecular and enzymatic antioxidants, with plant stress tolerance being strongly related to the activity of the antioxidant system of cells (Kraj & Slepaczuk, 2022; Sidhu et al., 2016). The growth of plants under stress conditions is part of their life, and determining the limits of their tolerance and acclimatization abilities to these conditions is of practical importance in predicting the adaptive potential of individuals (Isebrands et al., 2000).

The seedlings of some tree species can successfully develop in areas polluted by heavy metals. Research on these species is important in the context of exploring the potential for introducing tree species to achieve the permanent biological stabilization and reclamation of post-flotation tailings, especially after the final recycling of trace metals, but where concentrations remain much higher than in natural soils. To better understand the adaptation and reaction to plant stress induced by heavy metal ions of *Betula pendula* Roth., *Pinus sylvestris* L., and *Larix decidua* Mill. seedlings caused by tailings waste highly contaminated by trace elements, the relationships between: the concentration of heavy metals in the soil substrate, the ability to their accumulation, and the biometric parameters of the seedlings were determined. We also estimated the threshold content of heavy metals in the roots above which the oxidative burst, an early plant response to heavy metal content increase is triggered. We assume that there are certain limit concentrations of heavy metals in the soil and fine roots, which depend on the tree species and beyond which the plant responds strongly to stress. We believe that the tolerance limits and the ability of forest tree seedlings to acclimatize under stressful conditions depend on the species. Additionally, there are certain ranges of heavy metal concentrations in the soil at which the physiological response is the greatest.

## Materials and methods

### Study sites and field study

The experiment was carried out between March and October in the Olkusz Forestry Nursery, Poland. The average annual air temperature was 7.7°C and the average total precipitation during the experiment was 562 mm (the average annual precipitation was 741 mm). Stimulated contamination of the substrate by sludge taken from a post-flotation tailings ponds was used. Different concentrations of trace metals were obtained by mixing the nursery substrate with a volumetric ratio of post-flotation tailings taken from the tailing ponds (Table 1).

**Table 1:** Experimental substrate

Substrate	Volumetric share of nursery substrate	Volumetric share of flotation tailings
SA	100%	0%
SA+10%PFS	90%	10%
SA+25%PFS	75%	25%
SA+50%PFS	50%	50%

*SA* Nursery substrate; *PFS* flotation tailings

One-year-old seedlings of silver birch (*Betula pendula* Roth), Scots pine (*Pinus sylvestris* L.), and European larch (*Larix decidua* Mill.) were tested in the experiment. Light-demanding species were selected for the study, which when placed in an open area will tolerate strong sunlight well. Pine is one of the main forest-forming species in Europe and is characterized by low habitat requirements. Pine trees occur in various soil conditions with a wide range of humidity conditions. *Betula* trees have a shallow, extensive root system that can effectively absorb heavy metals from the soil. Moreover, *Betula* is often a succesional species. *Larix* has a strong tap root system with numerous lateral roots. *Larix* is characterized by very rapid growth when young. Each seedling was planted in a container with a capacity of 5 dm^3^. For each substrate variant and species, 10 seedlings were planted in March (120 seedlings in total). In June (date 1) and October 2021 (date 2), 60 seedlings were collected, in which the content of macro- and selected microelements (Cd, Pb, Zn) were analyzed.

### Laboratory analyses

In the substrate samples, pH was measured potentiometrically in 1 M potassium chloride (KCl). The N content was measured using the dry combustion method using a TruMac® CNS analyzer. The total contents of magnesium (Mg), (potassium) K, phosphorus (P), (cadmium) Cd, (lead) Pb, and (zinc) Zn were measured using inductively coupled plasma–optical emission spectroscopy (ICP–OES) (iCAP™ 6000 series) after extraction in a mixture of nitric (HNO_3_) and perchloric (HClO_4_) acids at a ratio of 3:1. Trace elements (Cd, Pb, Zn) were extracted using 1M ammonium acetate (CH_3_COONH_4_) and determined using ICP–OES.

After extraction from the substrate, the seedlings were transported to the laboratory and washed in distilled water. The cuttings were divided into roots, shoots, and leaves. The root systems were scanned and analyzed using WinRHIZO software, which allows determination of the total root length and average root diameter. The roots, shoots, and leaves were dried at 65°C and weighed to an accuracy of 0.01 g on a laboratory balance. The elements (Mg, K, P, Cd, Pb, and Zn) in the roots, stems, and leaves were measured using a spectrophotometer after wet combustion in a mixture of HNO_3_ and HClO_4_ at a ratio of 4:1. The total N content was measured using the dry combustion method and a TruMac analyzer.

### Oxidative stress marker determination

The intensity of oxidative stress caused by the uptake and accumulation of heavy metals was determined based on the content of H_2_O_2_ and the level of the biochemical marker of lipid peroxidation (TBARS) in the roots, shoots, and assimilation organs. The ability to scavenge the H_2_O_2_, and indirectly the ROS, transformed into this compound was determined by analyzing the total ascorbic acid (tAsA) content and the percentage of its oxidized form as dehydroascorbic acid (DHA).

Seedlings intended for the analysis of oxidative stress markers and the H_2_O_2_ scavenging rate were rinsed of substrate, divided into their organs (leaves/needles, shoots, and roots), frozen in liquid N, and then ground into powder. H_2_O_2_ content was determined using the Amplex Red oxidation method (Cayman Chemical, Ann Arbor, MI, USA), as described by Kraj, (2016). Briefly, H_2_O_2_ was extracted in 50 mM sodium phosphate buffer (pH 7.4) containing Triton X-100 (0.2%). The Amplex Red oxidation was carried out in the dark at 30°C for 30 min. The components of the reaction mixture (0.1 mM Amplex Red and 0.2 U/ml horseradish peroxidase (Sigma, St. Louis, MO, USA)) were dissolved in sodium phosphate buffer, and then this solution was mixed with the H_2_O_2_ extract in a 1:1 ratio. The absorbance was read at 560 nm, and the H_2_O_2_ concentration was determined from the standard curve for the range of 0–8 μM.

The degree of lipid peroxidation was determined by analyzing the TBARS content according to the method of Dhindsa et al. (1981). TBARS extraction was performed with 5% trichloroacetic acid (TCA). The reaction mixture containing the extract and a 20% solution of TCA plus thiobarbituric acid (TBA) (Sigma, St. Louis, MO, USA) was heated at 95°C for 30 min and then quickly cooled in ice. The TBARS concentration was calculated using an extinction coefficient of 155 mM^−1^ cm^−1^ based on the difference in absorbance at 532 nm and non-specific absorbance at 600 nm.

### Ascorbate and dehydroascorbate determination

The total content of ascorbic acid (tAsA) and the content of its reduced form were determined according to the method of Gillespie and Ainsworth, (2007). Ascorbic acid extract in 6% TCA was obtained. To analyze the total content of ascorbic acid, the oxidized form of this compound was reduced by adding 10 mM dithiothreitol (DTT). The solution was incubated at 37°C for 20 min. To remove DTT excess 0.5% N-ethylmaleimide (NEM) was added. The reduced form of ascorbic acid was determined without the DTT or NEM. A reaction mixture containing TCA, phosphoric acid (H_3_PO_4_), 2,2'-dipyridyl, and iron chloride (FeCl_3_) was added to the ascorbic acid extract. The reaction was carried out at 37°C for 60 min. The absorbance at 550 nm was read, and the content of ascorbic acid was determined on the basis of a standard curve plotted for concentrations of 0.15–10 mM. The content of the oxidized form of ascorbic acid (DHA) was calculated as the difference between the total and reduced forms of the compound and expressed as a % of its total content.

All absorbance readings were taken using a Microplate Reader Synergy-2 (Biotek, Winooski, VT, USA).

### Statistical procedures

Statistical analysis included the implementation of generalized additive models (GAMs). The GAM method with an identical binding function was used for prognostic calculations. One-way analysis of variance (ANOVA) was used to check the differences in mean values of the soil properties. Two-way ANOVA was used to check the differences in mean values of the biometric parameters, the total content of Mg, K, P, Cd, Pb, and Zn, and determine the oxidative stress marker. This was followed by a multiple comparison procedure using the RIR–Tukey test (at *p* < 0.05). The statistical analysis was performed using Statistica 13.3 software (StatSoft, Inc.).

## Results

### Basic properties of the substrates

The post-flotation tailings used to prepare the substrates were slightly alkaline (pH 7.61). The nitrogen (N) content was 0.009%, and the content of exchangeable cations was calcium (Ca^2+^) 7.99, potassium (K^+^) 0.01, magnesium (Mg^2+^) 0.87, and sodium (Na^+^) 0.02 all in cmol∙kg^−1^. The total concentration of Cd was 0.08 mg∙g^−1^, whereas the concentrations of Pb and zinc (Zn) were 1.96 and 8.23 mg∙g^−1^, respectively.

The substrates used in the experiment differed in their physicochemical properties. The SA substrate was very strongly acidic (pH 4.98), whereas the remaining substrates were neutral, the higher the PFS content (Table 1). The high pH is related to the properties of the ore-bearing rocks from which the sediments were formed. Zinc–Pb ores in Poland occur mainly in Middle Triassic dolomites, marly dolomites, ore-bearing limestones, and marly limestones, which, due to their high Ca and Mg carbonate (CO_3_) contents, affect the alkalization of the substrates. Most trace metals in this pH range show relatively low mobility (Krzaklewski & Pietrzykowski, 2002). The content of macronutrients in the substrates was inversely related to the percentage of PFS. The substrate without the addition of PFS had the highest N content (6.10 mg∙g^−1^), whereas the SA + 50% PFS substrate contained the least N (0.46 mg∙g^−1^). The decrease in N content in substrates containing a higher proportion of PFS resulted from the lack of this element in the post-flotation tailings. The N content in the post-flotation tailings taken from the tailing ponds was 0.09 mg·g^−1^. Similar results were obtained for Ca, K, Mg, and Na (Table 2). The content of heavy metals (Cd, Pb, Zn) increased with an increasing share of PFS in the substrate, except for Pb, the content of which was comparable with the substrates SA + 25% PFS and SA + 50% PFS (approximately 0.55 mg∙g^−1^) (Table 2).

**Table 2:** One-way ANOVA for the basic soil properties (mean ± standard errors)

Soil properties	Units	SA	SA+10% PFS	SA+25% PFS	SA+50% PFS
pH KCl		4.98±0.19^a^	7.05±0.14^c^	7.20±0.07^bc^	7.29±0.05^b^
N_tot_	mg g^−1^	6.1±0.19^a^	1.86±0.11^b^	1.07±0.08^c^	0.46±0.02^d^
C_tot_	mg g^−1^	372.76±11.78^a^	162.04±6.57^b^	126.36±4.87^c^	97.13±1.82^d^
Ca^2+^	cmol kg^−1^	29.74±1.59^a^	30.18±2.88^a^	19.57±1.17^b^	11.09±0.88^c^
K^+^	cmol kg^−1^	0.18±0.01^a^	0.08±0.01^b^	0.05±0.00^c^	0.02±0.00^d^
Mg^2+^	cmol kg^−1^	19.35±0.86^a^	8.65±0.79^b^	4.25±0.41^c^	1.35±0.29^d^
Na^+^	cmol kg^−1^	0.24±0.06^a^	0.08±0.02^b^	0.04±0.02^c^	0.02±0.01^c^
Cd	mg kg^−1^	0.26±0.05^d^	9.15±0.76^b^	14.07±0.75^a^	11.83±0.46^c^
Pb	mg kg^−1^	2.66±1^c^	263.58±12.29^b^	460.46±28.01^a^	475.99±28.11^a^
Zn	mg kg^−1^	6.67±1.24^c^	180.62±14.08^b^	235.92±12.56^a^	194.35±8.72^b^
Cd_tot_	mg kg^−1^	1.25±0.20^d^	55.08±0.63^c^	74.32±0.54^b^	80.56±2.41^a^
Pb_tot_	mg kg^−1^	126.4±13.24^b^	2274.5±65.40^a^	2407.7±106.98^a^	2456.1±166.85^a^
Zn_tot_	mg kg^−1^	217.4±21.87^d^	5716.3±71.10^c^	7015.4±55.60^b^	7399.3±91.99^a^

Different letters indicate significant differences between the variants substrate. *C*_*tot*_ Contents of carbon; *N*_*tot*_ contents of nitrogen; *Ca*^*2+*^ exchangeable calcium; *K*^*+*^ exchangeable potassium; *Mg*^*2+*^ exchangeable magnesium; *Na*^*+*^ exchangeable sodium; *Cd* exchangeable cadmium; *Pb* exchangeable lead; *Zn* exchangeable zinc, *Cd*_*tot*_ contents of cadmium; *Pb*_*tot*_ contents of lead; *Zn*_*tot*_ contents of zinc

### Biometric parameters and chemistry of the seedlings

The tested species differed in their shoot, root, and leaves biomass. *Betula* was characterized by having the lowest leaves biomass (0.9 g). By contrast, *Pinus* had the lowest shoot (1.7 g) and root biomass (1.0 g). The average root diameter was the smallest in the *Betula* seedlings (0.4 mm), larger in *Pinus* (0.5 mm), and largest in *Larix* (0.6 mm). The seedlings growing in the SA + 50% PFS variant were characterized by a shorter root length and a larger root diameter than the seedlings growing in the other variants. The average root length for the seedlings growing in the SA variant was 1274.4 mm, whereas, in the SA + 50% PFS variant, it was about 50% shorter, at 623.1 mm. The average root diameter for seedlings growing in the SA + 50% PFS variant was 0.7 mm, and 0.5 mm for seedlings growing in the other variants (Table 3).

**Table 3:** Biometric parameters of birch, larch and pine seedlings growing in the tested substrates

	Lenght roots [mm]			Avg diameter roots [mm]			Mass stem [g]			Mass roots [g]			Mass leaves [g]		
	*Betula*	*Pinus*	*Larix*	*Betula*	*Pinus*	*Larix*	*Betula*	*Pinus*	*Larix*	*Betula*	*Pinus*	*Larix*	*Betula*	*Pinus*	*Larix*
SA	1821.1±337^a^	1141.9±150.2^ab^	952±302.5^ab^	0.3±0.0^b^	0.5±0.0^a^	0.6±0.1^a^	3.0±1.2^a^	1.4±0.2^b^	3.0±0.8^a^	1.5±0.4^a^	0.9±0.2^b^	1.7±0.6^a^	0.7±0.2^b^	2.4±0.4^a^	2.9±0.4^a^
SA+10%PFS	1602.3±131.4^a^	1207.4±104.4^a^	544.5±180.1^b^	0.4±0.1^a^	0.5±0.0^a^	0.5±0^a^	3.3±0.5^a^	1.7±0.3^b^	2.5±0.6^ab^	1.7±0.3^a^	1.1±0.1^b^	1.2±0.5^b^	0.9±0.2^b^	2.4±0.4^a^	3.0±0.1^a^
SA+25%PFS	1139±123.6^a^	922.1±77.8^ab^	744±241.1^b^	0.4±0.1^b^	0.5±0.0^ab^	0.6±0.1^a^	2.7±0.7^ab^	1.9±0.2^b^	4.4±1.3^a^	1.2±0.3^b^	1.1±0.2^b^	1.9±0.5^a^	0.7±0.2^b^	2.6±0.2^a^	2.7±1.2^a^
SA+50%PFS	983.8±197.9^a^	489.1±53.7^b^	423.4±91.2^b^	0.5±0.1^b^	0.7±0.0^a^	0.7±0.1^a^	4.8±1.3^a^	1.9±0.4^b^	2.3±0.8^b^	1.8±0.5^a^	0.8±0.1^b^	0.9±0.3^b^	1.1±0.5^b^	2.7±0.4^a^	1.3±0.6^b^

Different letters indicate significant differences between the species

The N content in the *Betula* leaves was higher than in the *Pinus* and *Larix* needles. A higher content of N and K was found in the leaves of the seedlings growing in the SA variant. The N supply of the shoots did not differ across species. The seedlings growing in different test substrates did not differ in their content of P in the roots, shoots, or leaves. The supply of Mg to the leaves of the tested seedlings was the highest for *Betula* in the SA + 10% PFS and SA + 25% PFS variants (Table 4).

**Table 4:** Two-way ANOVA for the N, P, K, and Mg contents (mean values ± standard errors) in seedlings studied tree species growing on different substrates

		N [mg g^−1^]			P [mg g^−1^]			K [mg g^−1^]			Mg [mg g^−1^]		
		*Betula*	*Pinus*	*Larix*	*Betula*	*Pinus*	*Larix*	*Betula*	*Pinus*	*Larix*	*Betula*	*Pinus*	*Larix*
Roots	SA	6.4±0.3^bA^	7.3±0.3^aB^	5.1±0.0^b^	0.7±0.1^aA^	0.7±0.0^aA^	0.6±0.0^bA^	2.6±0.0^aA^	3.6±0.2^aA^	2.8±0.0^aA^	1.3±0.1^aA^	1.3±0.0^aA^	1.2±0.0^aB^
	SA+10%PFS	4.5±0.2^bB^	6.3±0.0^aC^	5.8±0.1^a^	0.5±0.0^aA^	0.5±0.0^aA^	0.5±0.0^aA^	2.6±0.0^aA^	2.8±0.1^aA^	2.7±0.2^aA^	1.3±0.0^bA^	1.7±0.0^aA^	1.6±0.2^aA^
	SA+25%PFS	6.1±0.4^aAB^	6.5±0.2^aC^	4.7±0.1^b^	0.6±0.1^aA^	0.5±0.0^aA^	0.6±0.0^aA^	2.9±0.2^aA^	2.8±0.0^aA^	2.9±0.2^aA^	1.4±0.1^bA^	1.7±0.1^aA^	2.0±0.1^aA^
	SA+50%PFS	5.6±0.3^bAB^	7.7±0.3^aA^	5.6±0.3^b^	0.6±0.1^aA^	0.6±0.1^aA^	0.7±0.0^aA^	2.7±0.2^aA^	3.0±0.4^aA^	2.9±0.2^aA^	1.4±0.0^bA^	1.9±0.2^aA^	1.9±0.1^aA^
Leaves	SA	18.0±0.0^aA^	11.5±0.3^bA^	10.6±0.0^bA^	1.1±0.0^aA^	0.6±0.1^bA^	0.7±0.0^bA^	6.2±0.0^aA^	4.4±0.3^bA^	4.7±0.0^bA^	3.8±0.0^aB^	1.6±0.1^cB^	2.2±0.0^bA^
	SA+10%PFS	13.8±0.0^aC^	8.7±0.1^bB^	9.9±0.0^bA^	1.1±0.0^aA^	0.5±0.0^bA^	0.7±0.0^bAB^	5.3±0.0^aAB^	2.9±0.2^bB^	3.0±0.0^bB^	4.7±0.0^aA^	1.5±0.1^cB^	2.0±0.0^bA^
	SA+25%PFS	15.9±0.0^aB^	8.3±0.3^bB^	9.5±0.0^bA^	0.9±0.0^aA^	0.5±0.0^bA^	0.9±0.0^aBC^	4.9±0.0^aAB^	3.2±0.3^bB^	3.3±0.0^bB^	4.7±0.0^aA^	1.5±0.1^cB^	1.9±0.0^bA^
	SA+50%PFS	16.5±0.0^aAB^	8.6±0.7^cB^	10.6±0.0^b^	1.0±0.0^aA^	0.6±0.1^bA^	1.3±0.0^aC^	4.3±0.0^aB^	2.5±0.3^bB^	3.5±0.0^abB^	4.0±0.0^aB^	1.9±0.1^bA^	2.1±0.0^bA^
Stems	SA	5.5±0.1^aA^	5.3±0.3^aAB^	6.0±0.6^aB^	0.5±0.0^aA^	0.5±0.0^aA^	0.5±0.0^aB^	2.6±0.3^bB^	3.6±0.1^aA^	3.5±0.0^aA^	0.9±0.0^bA^	1.4±0.0^aA^	1.1±0.0^bB^
	SA+10%PFS	5.5±0.0^aA^	4.0±0.1^aC^	6.9±1.5^bA^	0.5±0.0^bA^	0.4±0.0^bA^	0.8±0.1^aA^	2.2±0.1^bB^	3.1±0.2^aAB^	1.9±0.1^bC^	0.9±0.0^bA^	1.4±0.0.0^aA^	1.1±0.1^bB^
	SA+25%PFS	5.4±0.0^aA^	4.4±0.1^bBC^	4.1±0.7^cC^	0.5±0.0^aA^	0.4±0.0^aA^	0.5±0.1^aB^	2.5±0.0^bB^	2.7±0.2^aB^	2.8±0.6^aB^	0.9±0.1^bA^	1.3±0.1^aA^	1.0±0.1^bB^
	SA+50%PFS	4.4±0.1^aB^	5.5±0.1^bA^	5.8±0.7^cB^	0.4±0.0^bA^	0.5±0.0^bA^	0.8±0.1^aA^	1.9±0.1^bA^	3.2±0.2^aA^	2.6±0.3^abB^	0.7±0.0^bB^	1.3±0.1^aA^	1.3±0.2^aA^

Different letters (a, b, c, d) indicate significant differences between the studied species (for the same substrate). Different capital letters (A, B) indicate significant differences between the studied types of parent materials (on the same trees species)

The Cd, Pb, and Zn contents in the *Betula*, *Larix*, and *Pinus* seedlings depended on the tree species and the substrate. The highest concentration of cadmium was found in fine pine roots and birch leaves and shoots. The highest lead concentration was found in fine roots and pine shoots. The concentration of lead in pine and larch needles did not differ and was higher than the concentration of lead in birch leaves. The highest concentration of zinc was found in fine pine roots. The concentration of zinc was highest in birch shoots and leaves (Table 5). The bioaccumulation factors of Cd, Pb, and Zn by the roots, leaves, and shoots were, in most cases, lower than 0.1. Bioaccumulation factors above 1 were found in the roots of the seedlings grown in uncontaminated substrates

**Table 5:** Two-way ANOVA for the Cd, Pb, and Zn contents (mean values ± standard errors) in seedlings studied tree species growing on different substrates

		Cd [mg kg^−1^]			Pb [mg kg^−1^]			Zn [mg kg^−1^]		
		*Betula*	*Pinus*	*Larix*	*Betula*	*Pinus*	*Larix*	*Betula*	*Pinus*	*Larix*
Roots	SA	5.24±1.27^aB^	1.87±0.43^aC^	1.46±0.00^aC^	198.13±66.67^aD^	60.26±19.65^bD^	34.64±0^bD^	227.76±2.89^aC^	105.33±17.85^aC^	61.64±0.00^bC^
	SA+10%PFS	11.17±1.05^aB^	15±0.48^aB^	7.97±2.71^aBC^	573.88±39.88^bC^	1017.34±44.37^aC^	385.7±148.3^cC^	402.7±54.97^abBC^	514.99±48.39^aB^	282.97±63.72^bC^
	SA+25%PFS	22.12±0.35^aAB^	27.47±0.83^aB^	26.32±1.63^aAB^	1051.64±41.56^cB^	1657.06±31.53^aB^	1198.96±51.07^bB^	619.44±60.17^bAB^	780.39±62.25^aB^	642.17±28.22^abB^
	SA+50%PFS	31.16±0.42^bA^	53.44±6.15^aA^	36.11±2.78^abA^	1556.36±81.32^cA^	2794.31±38.57^aA^	1726.56±53.28^bA^	723.45±60.05^bA^	1310.94±17.22^aA^	946.37±28.83^cA^
Leaves	SA	0.74±0.00^aC^	0.79±0.16^aC^	0.27±0.00^aB^	25.38±0.00^aA^	35.75±9.53^aB^	10.5±0.00^aB^	134.04±0.00^aB^	99.01±11.46^aB^	27.54±0.00^aB^
	SA+10%PFS	5.09±0.00^aB^	1.83±0.25^bB^	1.34±0.00^bB^	69.27±0.00^aA^	62.21±11.67^aB^	52.87±0.00^aAB^	414.39±0.00^aA^	145.44±17.33^bB^	70.04±0.00^bB^
	SA+25%PFS	11.41±0.00^aA^	3.36±0.52^bB^	4.35±0.00^bA^	89.13±0.00^aA^	123.76±27.44^aB^	175.86±0.00^aAB^	458.34±0.00^aA^	201.37±35.88^bB^	131.29±0.00^bAB^
	SA+50%PFS	4.95±0.00^aB^	5.88±0.95^aA^	5.36±0.00^aA^	41.68±0.00^bA^	290.14±75.57^aA^	224.11±0.00^abA^	364.89±0.00^aA^	400.72±92.09^aA^	187.64±0.00^aA^
Stems	SA	0.68±0.12^aD^	1.17±0.14^aD^	0.87±0.15^aD^	8.07±1.56^bC^	52.34±6.27^aC^	33.91±6.96^aC^	155.97±20.38^aC^	116.8±12.85^aC^	61.44±10.33^bB^
	SA+10%PFS	4.39±1.3^aC^	3.21±0.44^bC^	2.13±0.21^bC^	96.87±53.05^aA^	69.85±16.55^bC^	59.55±17.21^bBC^	219.98±55.51^aB^	164.04±17.13^bB^	93.9±19.11^cB^
	SA+25%PFS	7.99±0.22^aA^	4.9±0.35^bB^	4.0±0.53^bB^	42.48±0.51^bB^	108.56±15^aB^	85.57±18.27^aB^	262.24±32.8^aA^	193.12±20.31^bB^	98.57±20.73^cB^
	SA+50%PFS	6.75±0.77^aB^	6.09±0.27^bA^	5.94±1.19^bA^	98.24±44.93^bA^	205.19±22.77^aA^	208.09±51.61^aA^	224.65±29.55^aB^	252.37±23.8^aA^	199.38±40.2^aA^

Different letters indicate significant differences between the variants substrate

The correlation coefficient between the concentration of trace elements (Pb, Cd, Zn) in the soil and the biometric parameters of the seedlings (leaves weight, shoot weight, root weight, root length, root diameter) revealed a significant negative correlation between the trace elements and the root length in the first period of growth (from the beginning of the experiment until June). The correlation coefficients between root length and Cd, Pb, and Zn were −0.48, −0.60, and −0.45, respectively. To better understand the effect of trace-element concentrations on root length using a GAM, a model was developed to explain the relationship between the soil concentration of Cd, Pb, and Zn and root length. An important factor influencing root length was the concentration of Cd, with an increase in Cd concentration from 0 to 0.015 mg∙g^−1^ causing a several-fold decrease in root length.

### Influence of heavy metal concentration on the level of oxidative stress

The intensity of oxidative stress caused by the uptake and accumulation of heavy metals in the organs (roots, shoots, and leaves/needles) of *Betula*, *Pinus*, and *Larix* seedlings, and their ability to scavenge ROSs, depended on the content of heavy metals (Cd, Pb, Zn) in the seedlings. The reaction of the plants to the time of growth in the substrates containing heavy metals was determined, taking into account the seedling species and organ. The seedling species differed in the degree of lipid peroxidation in the roots, shoots, and leaves/needles, and the content of H_2_O_2_ in the leaves/needles. A significantly higher TBARS content was found in the organs of *Larix* compared to those of the *Pinus* and *Betula* seedlings (Table 6).

**Table 6:** Contents of TBARS, H_2_O_2_ and the reduced form of ascorbic acid (tAsA) and the share of its oxidized form (%DHA) in the organs of birch, pine, and larch seedlings

	*Betula*		% changes	*Pinus*		% changes	Larix		% changes
	Term 1	Term 2		Term 1	Term 2		Term 1	Term 2	
Root TBARS	436.23	316.17	−27.52%	810.4	369.68	−54.38%	994.71	541.82	-45.53%
Shoot TBARS	277.13	218.39	−21.20%	445.2	389.66	−12.48%	623.26	334.68	-46.30%
Leaf TBARS	217.48			322.67	672.28	108.35%	348.51	1500.89	330.66%
Root H_2_O_2_	0.58	0.86	48.28%	0.3	1.35	350.00%	0.39	0.96	146.15%
Shoot H_2_O_2_	0.25	0.28	12.00%	0.27	0.13	−51.85%	0.22	0.4	81.82%
Leaf H_2_O_2_	0.09			0.23	0.69	200.00%	0.15	1.32	780.00%
Root tAsA	262.4	255.35	−2.69%	390.89	241.32	−38.26%	578.63	372.79	-35.57%
Shoot tAsA	91.97	71.74	−22.00%	203.42	128.96	−36.60%	111.56	86.08	-22.84%
Leaf tAsA	155.72			152.57	88.85	−41.76%	301.31	229.54	-23.82%
Root %DHA	0.38	0.44	15.79%	0.02	0.43	2050.00%	0.04	0.49	1125.00%
Shoot % DHA	0.1	0.48	380.00%	0.15	0.5	233.33%	0.08	0.49	512.50%
Leaf % DHA	0.18			0.15	0.49	226.67%	0.3	0.52	73.33%

The highest TBARS content was found with the lowest content of Cd*.* An increase in Cd content in the roots growing on substrates with an increasing share of flotation sludge resulted in a decrease in the degree of lipid peroxidation (Table 7). A different reaction in the roots was found for Zn and Pb, which caused either an increase or a constant content of TBARS, respectively, with an increase in the metal content. The remaining organs of the seedlings, except for Pb ions in the leaves/needles, did not show a clear relationship between the increasing content of metals and the level of lipid peroxidation (Table 7). The needles of the *Larix* seedlings showed a significantly greater increase in the H_2_O_2_ content in response to the uptake of heavy metals relative to the *Betula* seedlings, and a slightly smaller increase relative to the *Pinus* seedlings. The increasing Cd content in the *Larix* needles caused an increase in the H_2_O_2_ content (Table 7). At the same time, no other correlation was found between the increasing content of the other metals and the intensity of the oxidative stress.

**Table 7:** The influence of increasing contents of Cd, Zn, and Pb in the organs of *B. pendula*, *P. sylvestris*, and *L. decidua* seedlings on the content TBARS, H_2_O_2_, and the reduced form of ascorbic acid (tAsA) and the share of its oxidized form (%DHA)

Związek	Organ	Cd	Zn	Pb	*R*^2^
TBARS	Root	↘	↗	↔	75%
	Shoot	N	↔	N	72%
	Leaf	N	↔	↗	88%
H_2_O_2_	Root	↘	N	↗	65%
	Shoot	↗	↔	↔	71%
	Leaf	↗	N	↔	84%
tAsA	Root	↔	↔	↔	84%
	Shoot	↔	N	N	75%
	Leaf	↘	↗	↗	95%
% DHA	Root	↗	↔	N	69%
	Shoot	N	N	N	N
	Leaf	↗	↔	↔	94%

↗Content increase, ↘decrease in content, ↔no content changes, *N* negligible effect of the increase in metal content

Similarly to the markers of oxidative stress, there were significant interspecies differences in the markers indicating activity in the antioxidant system (i.e., tAsA) and the proportion of its oxidized form (% DHA) in the organs of the seedlings. The highest level of tAsA was found in the *Betula* seedlings, whereas significantly less was found in the coniferous *Pinus* and *Larix* seedlings. The tree seedlings growing on substrates containing heavy metals differed in their profiles of tAsA content change. It was found that all the organs of *Betula* seedlings were characterized by a continuous decrease in the content of this compound. By contrast, in response to the increasing content of heavy metals, the *Pinus* and *Larix* seedlings initially showed an increase in tAsA content, which reached its maximum in June, and then were characterized by a rapid decrease in this compound with increasing heavy metal content. The greatest decrease in tAsA during the vegetation period was found in the *Pinus* seedlings, with the decrease being less in the *Larix* and *Betula* seedlings (Table 6). In response to the oxidative stress caused by the presence of the heavy metals, the seedlings showed an increase in the share of the tAsA in its oxidized form (Table 6). The smallest increase in the share of DHA (relative to the tAsA) was found in the *Betula* organs. A significantly greater increase in the percentage of DHA was found in the organs of the *Larix* and *Pinus* seedlings. The roots of the coniferous seedlings showed a significantly higher degree of oxidation of ascorbic acid compared with the *Betula* seedlings. It was also found that the greatest increase in the share of DHA took place in the roots of the coniferous seedlings, with only a slight increase of this compound in the roots of the *Betula* seedlings. Significant differences were found in the concentrations of the markers of oxidative stress and forms of ascorbic acid in the organs of the *Betula*, *Larix*, and *Pinus* seedlings. These differences depended on the date of seedling sampling and how much time they had been growing on the substrates containing heavy metals. The TBARS content in the roots was significantly lower in September than in June for all three species. A similar tendency was found for the TBARSs in the shoots of the seedlings. However, the leaves showed a significant increase in the degree of lipid peroxidation in September, compared to the beginning of the vegetation period. The H_2_O_2_ content showed a significant increase in the roots and leaves/needles of the seedling species during the sampling period. While the increase in H_2_O_2_ content in the *Betula* roots was higher (by about 50%) in September than in June, its increase in the *Pinus* and *Larix* was several times higher than that (Table 6). The tAsA content showed a significant decrease in the roots of the seedlings. It was higher in the coniferous seedlings than in the *Betula* seedlings. The growth of the seedlings under stress conditions on the substrates containing heavy metals resulted in an increase in the share of the oxidized form of ascorbic acid in the organs of all the seedling species. The greatest increase in this share was in the roots of the coniferous tree seedlings, whereas it was significantly lower in their shoots and needles (Table 6).

## Discussion

The growth of the *Betula*, *Larix*, and *Pinus* seedlings was significantly inhibited in the contaminated substrates. One of the factors limiting their growth was the high concentrations of Pb, Zn, and Cd in the tested substrates. Kabata-Pendias & Pendias, (2001) reported that concentrations of 0.1–0.4 mg∙g^−1^ Pb and 0.07–0.4 mg∙g^−1^ Zn in soil are considered toxic to plants. Levy et al., (1999) showed that the phytotoxic concentration of Pb is 0.03–0.3 mg∙g^−1^, and above 0.1 mg∙g^−1^ for Zn. Differences in biomass reduction and root length reflect differences in the tolerance of seedlings to high concentrations of heavy metals in soils (Shi et al., 2011). The first signs of heavy metal toxicity in trees include a decrease in growth parameters, such as root elongation, root hair formation, and biomass production (Kahle, 1993). The seedlings of woody plants exposed to high concentrations of Cd have shown a reduction in root length (Arduini et al., 1994) or both root length and the root system biomass (Kahle, 1993; Lunackova et al., 2003). In a similar study, an increase in Cd concentration in the soil, from 0.59 to 2.4 mg∙kg^−1^, has been found to result in a 20% reduction in root length (Domínguez et al., 2009). In tree seedlings grown on contaminated soils, the rapid development of the root system is crucial for their survival, especially during periods of summer drought. In our study, the effect on root elongation was small at low or moderate levels of Cd in the soil, but a fourfold increase in the concentration of Cd caused a threefold reduction in the mass of the oak roots.

An important criterion for assessing the adaptability of forest tree seedlings to various habitat conditions is the physiological response of plants to the content of macroelements in their leaves (Lõhmus et al., 2006; Vares et al., 2004). Seedlings grown in the SA, SA + 10% PFS, and SA + 25% PFS variants were sufficiently supplied with N and Mg, while the substrate containing 50% PFS caused N, Mg, and K deficiency. A decrease in the supply of N and P, coupled with a simultaneous increase in the demand for these elements by plants, contributes to a reduction of the concentrations of these elements in the leaves (Lambers & Oliveira, 2019). Several authors have argued that the efficiency of nutrient use increases with decreasing nutrient availability in the soil. The lower concentration of P in the leaves, relative to optimal conditions, is probably due to the high pH of the growth medium and its consequent low availability for plants. The chemical composition of the leaves of *Pinus* and *Betula* seedlings grown on alkaline soils has been found to be 2.12% N, 0.18% P, 0.97% K, and 0.51% Mg for *Betula*, and 1.15% N, 0.11% P, 0.74% K, and 0.20% Mg for *Pinus* (Kuznetsova et al., 2010).

Metal accumulations in roots, shoots, and leaves are significantly lower than other trace-element hyperaccumulators (Shi et al., 2011). The metal contents in the tested seedlings may have result mainly from the period of growth in the contaminated soil (4 and 8 months). The high concentration of heavy metals in the roots indicates a high efficiency in immobilizing heavy metals, and a significant phytostabilization potential of the tested forest tree seedlings (Mertens et al., 2004). The bioaccumulation index––the ability of plants to accumulate heavy metals––was below 1 for most of the variants in our study, and the translocation of metals from the roots to the shoots and leaves seems to have been strongly limited. The roots accumulated a much higher content of heavy metals than the shoots and leaves, which indicates both the high availability of the metals and limited mobility inside the plant (Deng et al., 2004; Fitzgerald et al., 2003). The efficiency of heavy metal accumulation is reflected in the values of the bioaccumulation index, depending on the plant species, their tolerance to high concentrations of trace metals, and the concentrations of the trace metals in the soil (Jeyakumar et al., 2010; Pourrut et al., 2011). Our results are similar to the values obtained for other forest tree species occurring in polluted areas, where the bioaccumulation factor of the heavy metals was also determined to be below 1 for shoots and leaves (Brunner et al., 2008; Mertens et al., 2004; Nadgórska-Socha et al., 2017; Sebastiani et al., 2004). It is normally assumed that the values of the bioaccumulation index for a given metal are above 1 in plants suitable for phytoextraction, but that values below 1 are tolerable for the phytostabilization of heavy metals (Bech et al., 2012; Pourrut et al., 2011). The limited translocation of metals from roots to shoots suggests that shoot harvesting would not effectively remove metals from contaminated tailings (Shi et al., 2011).

The most important and common effect of the uptake of heavy metal ions by plants, including Cd, Pb, and Zn ions, and a symptom of their toxicity, is the formation of free radicals and ROSs, such as superoxide ions, H_2_O_2_, and lipid peroxidation products. This causes cell redox imbalance, oxidative stress, lipid peroxidation, and an increase in membrane leakage (Ashraf et al., 2019; Sandalio et al., 2001). In this study, it was confirmed by the increase in the content of oxidative stress markers (i.e., TBARSs and H_2_O_2_ in the roots, shoots, and leaves of the seedlings). The highest accumulation of heavy metals in the roots during the initial period of seedling growth (June) on the contaminated substrates resulted in the highest intensity of oxidative stress. As reactive particles become more abundant in plant cells, the cellular antioxidant system is activated, the task of which is to scavenge free radicals and ROSs and keeping their content at an appropriate level (Dubey et al., 2018; Kraj & Zarek, 2021; Singh et al., 2009). Although the formation of ROSs and their accumulation above physiologically acceptable levels are harmful, at low concentrations, these particles play a signaling role and activate the antioxidant system (Dubey et al., 2018). One of the main water-soluble cellular antioxidants is ascorbic acid (Kraj, 2016; Noctor & Foyer, 1998). Its basic function is to maintain the redox state of cells and protect them against stress factors by capturing H_2_O_2_ formed under the influence of stress factors, including the presence of heavy metals. The Foyer–Halliwell–Asada cycle plays a special role in maintaining an appropriate content of H_2_O_2_ in cells (Foyer et al., 1994; Noctor & Foyer, 1998), and its operation depends not only on the activity of APX––one of the enzymes catalyzing the decomposition of H_2_O_2_––but also on the tAsA content and the share of its reduced form (DHA) (Bielen et al., 2013). Therefore, decreasing the ascorbate content and increasing the ratios of its oxidized to reduced forms are often used as indicators of oxidative stress (Kukavica & Jovanovic, 2004).

Differences in the TBARS content in the seedlings’ organs between the initial (June) and final (September) growth periods testify to the gradually decreasing intensity of lipid peroxidation in the roots, while the intensity of this process increased in the leaves. The migration of heavy metals to the aboveground parts of the seedlings resulted in a species-dependent increase in damage to the leaves cell membranes. In contrast to changes in the cell membranes, the accumulation of H_2_O_2_ significantly increased both in the leaves and roots of the seedlings, especially in *Larix*. The increase in H_2_O_2_ content was significantly influenced not only by the uptake of metal ions, but also by their negative effect on the tAsA content and the increasing share of its oxidized form, which was not regenerated under stress conditions on the substrates containing harmful Cd, Pb, and Zn ions due to oxidative stress. This resulted in a deficiency of the reduced form of ascorbic acid, which is necessary as a substrate in the Foyer–Halliwell–Asad cycle that reduces the levels of H_2_O_2_ in cells (Bielen et al., 2013).

## Summary

The results of this study showed that *Betula* seedlings are suitable for the phytostabilization of post-flotation tailings due to their faster growth rate and higher production of root biomass than the seedlings of *Pinus* and *Larix*. Moreover, the quantity of metals accumulated in the *Betula* roots was much higher. Despite the high concentrations of heavy metals in the prepared substrates, the transfer of these elements to the aboveground parts of the plants was only slight. The correlation coefficient between the concentration of trace elements in the soil (Pb, Cd, Zn) and the biometric parameters of the seedlings (weight of the leaves, shoots, and roots; weight, length, and diameter of the roots) showed a significantly negative correlation between trace elements and root length in the first period of growth (from the beginning of the experiment until June). An important factor affecting root length was the concentration of Cd in the soil, with an increase causing a several-fold decrease in root length. The high concentration of heavy metals in the soil adversely affects the biochemical and physiological processes in plants and causes a reduction in the activity of the antioxidant system, depending on the species and organ as well as the type and concentration of metals. The conducted research has shown that in order to effectively bioremediate contaminated areas, it is necessary to remove entire plants, because the roots are mainly responsible for the bioaccumulation of heavy metals. Restoring stable forest ecosystems in urbanized areas will be important in the context of ongoing environmental degradation, and roots may act as a key indicator of the level of environmental pollution. The research results may be the basis for selecting appropriate tree species for use in the remediation and phytostabilization processes of heavily polluted post-industrial facilities.
